# Supplementary material for: Causal Relationship between Aging and Anorexia Nervosa: A White-Matter-Microstructure-Mediated Mendelian Randomization Analysis
Source: Biomedicines. 2024 Aug 16;12(8):1874. doi: 10.3390/biomedicines12081874 (PMC11351342; doi:10.3390/biomedicines12081874)
Supplement: Supplementary file 1 [file biomedicines-12-01874-s001.zip › Supplementary Figure/Figure S7/Single SNP plot of MR analysis for ubm-b-1943 to AN.pdf]

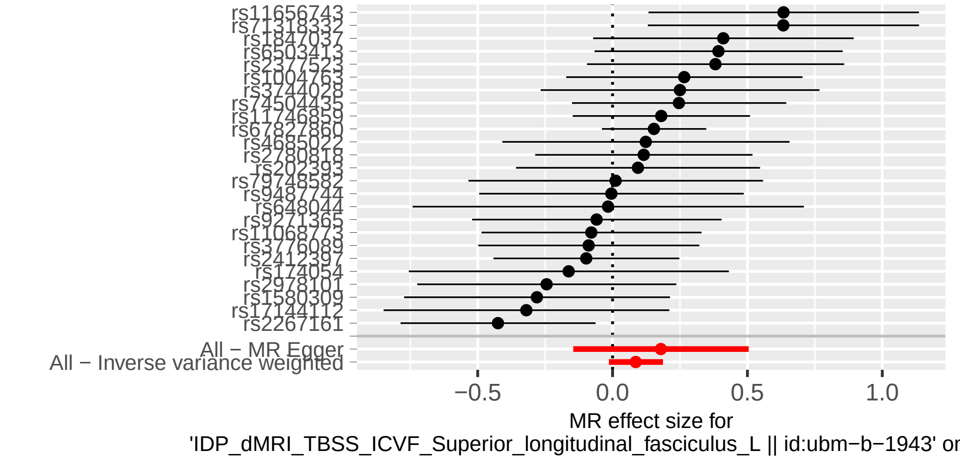

rs11656743
rs71318332
rs1847037
rs6503413
rs2377523
rs1004763
rs3744028
rs74504435
rs11746859
rs67827860
rs4685022
rs2780818
rs202393
rs79748582
rs9487744
rs648044
rs9271365
rs11068773
rs3776089
rs2412397
rs174054
rs2978101
rs1580309
rs17144112
rs2267161
All − MR Egger
All − Inverse variance weighted
−0.5
0.0
0.5
1.0
MR effect size for
'IDP_dMRI_TBSS_ICVF_Superior_longitudinal_fasciculus_L || id:ubm−b−1943' or
